# Supplementary material for: Emergent biaxiality in chiral hybrid liquid crystals
Source: Nat Commun. 2024 Nov 16;15:9941. doi: 10.1038/s41467-024-54236-8 (PMC11569233; doi:10.1038/s41467-024-54236-8)
Supplement: Supplementary file 1 — Supplementary Information [file 41467_2024_54236_MOESM1_ESM.pdf]

Supplementary Information
Emergent biaxiality in chiral hybrid liquid crystals

J.-S. Wu,[1] M. Torres Lázaro,[2] H. Mundoor,[1] H. H. Wensink,[2] and I. I. Smalyukh[1,3,4,5]

[1]Department of Physics and Chemical Physics Program, University of Colorado, Boulder, CO, USA
[2]Laboratoire de Physique des Solides - UMR 8502, Université Paris-Saclay & CNRS, 91405 Orsay, France
[3]International Institute for Sustainability with Knotted Chiral Meta Matter, Hiroshima University, Higashihiroshima, Japan
[4]Department of Electrical, Computer, and Energy Engineering, Materials Science and Engineering Program and Soft Materials Research Center, University of Colorado, Boulder, CO, USA
[5]Renewable and Sustainable Energy Institute, National Renewable Energy Laboratory and University of Colorado, Boulder, CO 80309, USA

I. BIAXIAL COLLOIDAL GAS-LIQUID COEXISTENCE OF HOMEOTROPIC RODS IN A CHOLESTERIC HOST

Let us focus on the case of rods with homeotropic anchoring conditions in a chiral molecular host. The total free energy acting on the rod Eq. (2) combines a surface anchoring and an elastic term which is given by the interpolated form Eq. (4). We ignore the tilt contribution which is much weaker than the disclination contribution Eq. (35) and focus on strong surface anchoring realignment so that rod points perpendicular to the nematic director $\hat{\mathbf{n}}$. The total free energy per rod may then be approximated as

$$F_{\mathrm{s,tot}} \sim \frac{\pi}{4} W_0 L_{\mathrm{c}} D_{\mathrm{c}} \gamma^2 + \Delta F_{\mathrm{twist}} \sin^2 \eta, \tag{36}$$

with γ the meridional angle with respect to the local nematic director $\hat{\mathbf{n}}$ and η an azimuthal angle so that $\gamma = 0$ and $\eta = 0$ corresponds to the rod pointing along the $\hat{\boldsymbol{\tau}}$-axis (Fig. 6). From this we define an orientational distribution function which in the general case of many interacting rods reads [1]

$$f(\gamma, \eta) = N \exp\left(-\tfrac{3}{2}\Gamma\gamma^2\right) \exp\left[-\Xi (qL_{\mathrm{c}})^2 \sin^2 \eta\right]. \tag{37}$$

The normalization factor is easily obtained $N = \sqrt{3\Gamma/(2\pi)^3} e^X / I_0(X)$ with $X = \Xi(qL_{\mathrm{c}})^2/2$ in terms of a modified Bessel function I_0. Here, Γ and Ξ represent two energy scale quantifying the typical surface anchoring and elastic energies in units of the thermal energy defined as

$$\begin{aligned} \Gamma &= -\frac{5}{4} c_r S_{\mathrm{c}} + \frac{\pi}{6} \beta W_0 L_{\mathrm{c}} D_{\mathrm{c}} \\ \Xi &= \frac{\pi}{12} \beta \Delta K L_{\mathrm{c}} \ln\left(\frac{2\ell_{\max}}{D_{\mathrm{c}}}\right), \end{aligned} \tag{38}$$

with $c_r = \frac{\pi}{4} N_{\mathrm{c}} L_{\mathrm{c}}^2 D_{\mathrm{c}}/V = \phi_r \ell_{\mathrm{eff}}$ a dimensionless rod concentration that related to the effective rod volume fraction ϕ_r with $\ell_{\mathrm{eff}} = 1536$ an effective rod aspect ratio that accounts for the electrostatic repulsion between the colloids (details are given in Ref. [1]). In the experimental situation, both energies are very large ($\Gamma = \mathcal{O}(10^2)$ and $\Xi = \mathcal{O}(10^3)$) and surface anchoring realignment completely dwarfs the alignment due to rod (excluded volume) correlations ($\beta W_0 L_{\mathrm{c}} D_{\mathrm{c}} \gg c_r |S_{\mathrm{c}})|$) so that in good approximation $\Gamma \approx \frac{\pi}{6} \beta W_0 L_{\mathrm{c}} D_{\mathrm{c}} \gg 1$. Following the definitions in Ref. [1] we obtain the uniaxial and biaxial order parameters as

$$\begin{aligned} S_{\mathrm{c}} &\sim \frac{1 - \Gamma}{2\Gamma}, \\ \Delta_{\mathrm{c}} &\sim I_1\left[\tfrac{1}{2}\Xi(qL_{\mathrm{c}})^2\right] / I_0\left[\tfrac{1}{2}\Xi(qL_{\mathrm{c}})^2\right]. \end{aligned} \tag{39}$$

In the absence of chirality $q = 0$ we find no biaxiality since $S_{\mathrm{c}} \approx -1/2$ and $\Delta_{\mathrm{c}} = 0$. However, even for weakly chiral systems with $qL_{\mathrm{c}} \ll 1$ we find a massive increase of the biaxial order parameter Δ_{c} *at the single colloid level* widely exceeding what is expected based on the Priest-Lubensky scenario that was envisaged for purely molecular chiral liquid crystals [2]. Indeed, for weak chirality we find up to quadratic order in the inverse pitch

$$\begin{aligned} \Delta_{\mathrm{c}} &\sim \Delta_0 (qL_{\mathrm{c}})^2 \sim \frac{\Xi}{4}(qL_{\mathrm{c}})^2 \\ &\propto \beta \Delta K q^2 L_{\mathrm{c}}^3, \end{aligned} \tag{40}$$

which demonstrates a pronounced cubic scaling with the colloid length $L_{\rm c}$. For rods with $L_{\rm c} = 1.7\mu$m and elastic anisotropy $\Delta K = 4$pN we find $\Delta_0 = \Xi/4 \approx 512$ which is about five orders of magnitude larger than the scaling amplitudes for the case of homeotropic disks which is based on the RP surface anchoring alone with the elastic distortions around the colloid negligible.

Let us now focus on biaxial fluids at elevated colloid concentrations ($c_r \gg 1$) where correlations among rods play a role. Then the free energy is given by the usual Onsager form [1, 3, 4]

$$F = \log c_r - 1 + \langle \ln f \rangle_f + \langle F_{\rm s,tot} \rangle_f + c_r \rho, \tag{41}$$

where the first two terms respectively account for the ideal and orientation entropy, the third contribution expresses the coupling with the molecular LC environment whereas the last term denotes the second-virial coefficient that accounts for volume exclusion between rods on the pair level. If the alignment is not too strong the angular dependence of the excluded volume between two rods labelled '1' and '2' can be expanded in terms of symmetry-adapted functions [5] and depends on the meridional and azimuthal angles via

$$\rho = \frac{4}{\pi} |\hat{\mathbf{u}}_1 \times \hat{\mathbf{u}}_2| \approx 1 - \frac{5}{8} \left[\mathcal{P}_2(\gamma_1)\mathcal{P}_2(\gamma_2) + \frac{1}{12}\mathcal{P}_2^2(\gamma_1)\mathcal{P}_2^2(\gamma_2)\cos(2(\eta_2 - \eta_1)) \right], \tag{42}$$

in terms of a second-order Legendre polynomial $\mathcal{P}_2$ and its associate $\mathcal{P}_2^2$. At infinite dilution $c_r \to 0$ the excluded-volume term in Eq. (41) becomes irrelevant and minimization with respect to the rod orientation distribution f yields the single-rod distribution given by Eq. (37). For the many-rod system we find a form similar to Eq. (37), namely

$$f(\gamma, \eta) = N e^{-\frac{3}{2}\Gamma\gamma^2} \exp\left[-(\Xi(qL_{\rm c})^2 + 2G)\sin^2\eta \right], \tag{43}$$

but with an additional concentration-dependent factor in the second exponent given by

$$G = \frac{5\pi}{48} c_r (1 - S_{\rm c})^2 \Delta_{\rm c}. \tag{44}$$

The normalization is given by the expression below Eq. (37) with $X = \Xi(qL_c)^2/2 + G$. The excluded-volume entropy can be expressed in terms of the orientational order parameters Eq. (39) and is given by

$$\rho \approx 1 - \frac{5}{8}\left[S_{\rm c}^2 + \frac{1}{3}(1 - S_{\rm c})^2 \Delta_{\rm c}^2 \right]. \tag{45}$$

From the free energy we easily extract the osmotic pressure $P = -(\partial F/\partial V)_{NT}$ and chemical potential $\mu = (\partial F/\partial N)_{VT}$ of the fluid

$$\begin{aligned} \beta P L_{\rm c} D_{\rm c}^2 &= c_r + c_r^2 \rho \\ \beta\mu &= \log c_r + \langle \ln f \rangle_f + 2c_r\rho + \langle F_{\rm s,tot}\rangle_f. \end{aligned} \tag{46}$$

The remaining averages pertaining to the orientational entropy and surface anchoring and elastic effects are also easily computed

$$\begin{aligned} \langle \ln f \rangle_f &= -\ln\left[2\pi I_0(\tfrac{1}{2}\Xi(qL_{\rm c})^2) + G \right]^{-1} + (\tfrac{1}{2}\Xi(qL_{\rm c})^2 + G)\Delta_{\rm c} + \frac{1}{2}\ln\left(\frac{3\Gamma}{2\pi} \right) - \frac{1}{2} \\ \langle F_{\rm s,tot} \rangle_f &= \tfrac{1}{2}\Xi(qL_{\rm c})^2(1 - \Delta_{\rm c}) + \frac{\pi}{12\Gamma}\beta W_0 L_{\rm c} D_{\rm c}. \end{aligned} \tag{47}$$

At a critical concentration rod correlations will promote strong biaxial order even in the absence of chirality [1]. To describe the strongly correlated biaxial regime we follow the analysis in Ref. [4] and approximate the orientational distribution by a Gaussian describing angular fluctuations of strength ω in the plane spanned by $\hat{\boldsymbol{\tau}}$ and $\hat{\boldsymbol{\chi}} \perp \hat{\mathbf{n}}$ but ignores excursion along the director $\hat{\mathbf{n}}$. The result is a product of two Gaussian angular distributions

$$f_G(\gamma, \eta) \sim \mathcal{N} e^{-\frac{3}{2}\Gamma\gamma^2} e^{-\frac{1}{2}\omega\eta^2}, \tag{48}$$

with normalization $\mathcal{N} = \sqrt{3\Gamma/(2\pi)^3}\sqrt{\omega/2\pi}$. In this case the principal ingredients of the free energy are given by the leading order asymptotic averages valid for $\omega \gg 1$. Ignoring all contributions smaller than $\mathcal{O}(\omega^{-1/2})$ we find

$$\begin{aligned} \rho = \frac{4}{\pi}\langle\langle |\hat{\mathbf{u}}_1 \times \hat{\mathbf{u}}_2| \rangle\rangle_{f_G} &\sim \frac{8}{\sqrt{\pi^3\omega}} \\ \langle \ln f_G \rangle_{f_G} &\sim \frac{1}{2}\ln\left[\frac{3\Gamma\omega}{(2\pi)^2} \right] - {\rm cst} \\ \langle F_{\rm s,tot} \rangle_f &\sim \frac{\pi}{12\Gamma}\beta W_0 L_{\rm c} D_{\rm c}. \end{aligned} \tag{49}$$

The constant featuring in the orientational entropy equals minus one if the angular fluctuations are strictly confined to the $\hat{\boldsymbol{\tau}} - \hat{\boldsymbol{\chi}}$ plane but we will here set it to a higher value cst ≈ 1.75 to partially account for weak angular fluctuation along the director $\hat{\mathbf{n}}$ and to obtain more realistic predictions of the coexistence densities for the achiral uniaxial-biaxial transition reported in Ref. [1]. Minimization of the free energy with respect to ω yields a well-known quadratic dependence of the angular fluctuation strength on the rod concentration $\omega \sim 64c_r^2/\pi^3$. The biaxial order parameter is correlated to ω via $\Delta_c \sim 1 - 2/\omega$.

Both ω and Δ_c are measures of biaxial order and both are monotonically increasing functions of the rod concentration c_r and chirality qL_c. Phase coexistence between different biaxial fluids can be established from equating the osmotic pressure and chemical potential in both phases. The benchmark case at zero chirality produces a uniaxial-to-biaxial phase transition discussed in Ref. [1]. At non-zero chirality the transition is strongly affected by the chiral disclination effect which gives rise to anomalous biaxial order as demonstrated in Fig. 2. Most notably, the phase diagram features an isostructural transition between two orthorhombic (biaxial) phases that closes off at a critical point at weak but finite chiral strength ($qL_c \approx 0.014$). Above the critical point only a single biaxial colloidal fluid state is found. We remark that the analysis presented in this Supplementary is only a crude version that merely serves to illustrate the main qualitative points of the phase diagram. The theory could be improved, for instance to get a better rendering of the near-critical region, by refining the description of the orientational distribution beyond the Gaussian approximation, for instance, by using a self-consistent form which needs to be implemented numerically [6].

II. CHIRALITY-RELATED CORRECTIONS FOR SURFACE ANCHORING AND SURFACE ELASTICITY

Weak director distortions enveloping around the rod cross-section (Fig. 3**c**) will also affect the surface anchoring energy with deviations expected from the RP predictions presented in the Methods. Similar to the elastic contribution we may analyze the specific effect of director twist on these corrections. Starting from the last term in Eq. (25) and parameterizing the surface normal vector $\hat{\mathbf{v}}(\mathcal{S}) = \cos\vartheta\hat{\mathbf{x}} + \sin\vartheta\hat{\mathbf{y}}$ in terms of the azimuthal angle ϑ we expand the surface anchoring free energy reads up to quadratic order in $qL_c \ll 1$

$$\frac{F_s}{L_c} = -\frac{W_0}{2}\oint_{\mathcal{C}} d\vartheta \left\{ \cos^2(\vartheta - \Phi) - \frac{(qL_c)^2}{12}\cos[2(\vartheta - \Phi)] \right\}, \tag{50}$$

where $\mathcal{C}$ denotes the circular contour of the rod cross-section with diameter D_c. For weak distortions $\Phi \ll 1$ we linearize for Φ and obtain

$$\frac{F_s}{L_c} \approx \frac{F_s^{(0)}}{L_c} - \frac{W_0}{2}(1 - \tfrac{1}{6}(qL_c)^2)\oint_{\mathcal{C}} d\vartheta \sin 2\vartheta\Phi. \tag{51}$$

The first term is the contribution for the *undistorted* director field previously analyzed

$$\begin{aligned} F_s^{(0)} &= -\frac{L_c W_0}{2}\oint_{\mathcal{C}} d\vartheta \left\{ \cos^2\vartheta - \frac{(qL_c)^2}{12}\cos 2\vartheta \right\} \\ &\sim -\frac{\pi}{4} W_0 L_c D_c, \end{aligned} \tag{52}$$

which corresponds to Eq. (23) for a homeotropic rod aligned perpendicular to the helical axis ($\theta = \delta = \pi/2$) in the weak chirality limit $qL_c \ll 1$. The change in RP surface anchoring free energy associated with a twist of the director distortions is as follows

$$\Delta F_{\text{twist}}^{(s)} \sim -\frac{\pi W_0 L_c D_c}{92}\frac{D_c}{\ell_s}(qL_c)^2, \tag{53}$$

which is only a fraction of the thermal energy for the previously defined experimental values for the surface anchoring amplitude W_0, extrapolation length ℓ_s and rod dimensions. We thus conclude that the twist of the LC host director leads to a marginal reduction of the surface anchoring energy which is dwarfed by a simultaneous increase of the elastic energy.

In order to gauge the effect of the finite core size of the colloidal rods, we consider the saddle-splay surface elasticity contribution which reads [7]

$$F_{se} = -\frac{K_{24}}{2}\int d\mathbf{r}\nabla \cdot (\hat{\mathbf{n}}_h \nabla \cdot \hat{\mathbf{n}}_h + \hat{\mathbf{n}}_h \times \nabla \times \hat{\mathbf{n}}_h). \tag{54}$$

with K_{24} the saddle-splay modulus.Expanding for weak twist and using the dipolar solution Eq. (34) to express the free energy change associated with saddle-splay elasticity upon twisting the surface defect we find after elaborate rearrangements

$$\Delta F_{\text{twist}}^{se} \sim \frac{7\pi}{12288} K_{24} L_{\text{c}} (q L_{\text{c}})^2 \left(\frac{D_{\text{c}}}{\ell_s} \right)^2 . \tag{55}$$

Taking $K_{24} = 3$pN we infer from the above expression that the saddle-splay elasticity is only a tiny fraction of the thermal energy and is thus of negligible importance for the colloidal rods considered in our study because of their vanishing thickness ($D_{\text{c}} \ll L_{\text{c}}$).

III. ELASTIC DISTORTIONS AROUND THE ROD SURFACE FOR $\hat{\mathbf{u}} \perp \hat{\boldsymbol{\chi}}$

In order to complete our understanding of the strength of the elastic distortions surrounding the main section of a thin rod we now focus on the case where a rod is perpendicular to the helical axis $\hat{\boldsymbol{\chi}}$ and aligned at an angle γ away from the $\hat{\boldsymbol{\tau}}$-axis. This situation is depicted in Fig. 7(a). Since the rod is perpendicular to the helical axis $\hat{\boldsymbol{\chi}}$ we may ignore the effect of chiral twist and parameterize the host director field case within a Cartesian reference frame spanned by the tripod $(\hat{\mathbf{x}}, \hat{\mathbf{y}}, \hat{\mathbf{z}})$ with $\hat{\mathbf{z}} = \hat{\boldsymbol{\chi}}$

$$\hat{\mathbf{n}}_h(\mathbf{r}) = \hat{\mathbf{x}} \cos \Phi(\mathbf{r}) \cos \varepsilon(\mathbf{r}) + \hat{\mathbf{y}} \sin \Phi(\mathbf{r}) \cos \varepsilon(\mathbf{r}) + \hat{\mathbf{z}} \sin \varepsilon(\mathbf{r}). \tag{56}$$

As before, we ignore distortions around the rod tips and express the spatial variation in terms of the distortion angles $\Phi(\mathbf{r})$ and $\varepsilon(\mathbf{r})$. In principle, the Euler-Lagrange expressions emerging from minimizing the elastic free energy are strongly coupled and cannot be solved analytically even in the case of weak surface anchoring.

In order to render our model analytically tractable we consider a much simpler set-up by assuming that the rod cross-section along which director distortions are expected to occur is curvature-free and can be described by an infinitely thin rectangular strip of length L_c and width $D_{\text{c}} \ll L_{\text{c}}$. The long axis of the strip points along $\hat{\mathbf{u}}$ while the short one (perpendicular to $\hat{\mathbf{u}}$) points along $\hat{\mathbf{z}} = \hat{\boldsymbol{\chi}}$. Next, we introduce the 2D Cartesian coordinates $\{r_n, z\}$ to parameterize the bulk LC deformation angle $\varepsilon(r_n, z)$ around the strip. The elastic free energy within the one-constant approximation with modulus K is as follows

$$F_{el} = \frac{K}{2} \iint dr_n dz \left\{ (\partial_z \varepsilon)^2 + (\partial_{r_n} \varepsilon)^2 \right\} \tag{57}$$

Here, $r_n \in [0, \infty]$ denotes the distance away from the strip along the surface normal vector $\hat{\mathbf{v}}$ whereas $z \in [0, D_{\text{c}}]$ parameterizes the distance across the short dimension of the strip along $\hat{\boldsymbol{\chi}}$. The distortion angle that minimizes the elastic free energy follows from the 2D Laplacian

$$(\partial_{r_n}^2 + \partial_z^2)\varepsilon = 0. \tag{58}$$

for which the general solution reads

$$\varepsilon(r_n, z) = \sum_{n=1}^{\infty} e^{-n\pi r_n} [a_n \cos(n\pi z) + b_n \sin(n\pi z)], \tag{59}$$

which vanishes in the far-field limit $\varepsilon(r_n \to \infty, z) = 0$ away from the strip surface normal. Next, we define a tilt angle $\gamma = \delta - \frac{\pi}{2}$ so that $\gamma = 0$ corresponds to the rod pointing along $\hat{\boldsymbol{\tau}}$ and $\gamma = \pi/2$ to the rod aligning along $\hat{\mathbf{n}}$.

The RP surface anchoring free energy given by Eq. (15) where the surface integral runs over the rectangular strip surface $\int d\mathcal{S} = L_{\text{c}} \int_0^{D_{\text{c}}} dz$. Furthermore, for an undistorted bulk LC the angle between the director field at the strip surface and the surface normal would simply be γ so that $\hat{\mathbf{n}}_h \cdot \hat{\mathbf{v}} = \cos\gamma$. For small director distortions $|\varepsilon| \ll 1$ near the strip surface it is reduced to $\gamma - \varepsilon$ and the dot product becomes $\hat{\mathbf{n}}_h \cdot \hat{\mathbf{v}} \approx \cos(\gamma - \varepsilon)$. The (dimensionless) surface anchoring energy then becomes

$$\frac{F_s}{K L_{\text{c}}} \approx -\frac{1}{2\ell_s} \int_0^{D_{\text{c}}} dz \cos^2(\gamma - \varepsilon(0, z)), \tag{60}$$

with $\ell_s \ll D_{\text{c}}$ the surface extrapolation length which is much larger than the rod diameter. The expression above translates into the following boundary condition at the surface of the strip located at $r_n = 0$

$$\partial_{r_n} \varepsilon(0, z) = \frac{1}{4\ell_s} \sin[2(\gamma - \varepsilon(0, z))]. \tag{61}$$

For symmetry reasons we require the distortion angle to be vanishing at both long edges of the strip $\varepsilon(0,0) = \varepsilon(0,D_{\rm c}) = 0$ which implies that $a_n = 0$. The coefficients b_n need to be resolved from

$$\frac{n\pi b_n}{2} = \frac{1}{4\ell_s}\int_0^{D_{\rm c}} dz \sin(n\pi z)\sin\left[2\left(\gamma - \sum_{k=1}^{\infty} b_k \sin(k\pi z)\right)\right]. \tag{62}$$

For small tilt angles $\gamma \ll 1$ distortions are expected to be weak $\varepsilon \ll 1$ so that we linearize $\sin 2(\gamma-\varepsilon) \approx 2(\gamma-\varepsilon)$. This enables us to resolve the coefficients analytically

$$b_n = \left(\frac{1-(-1)^n}{(n\pi)^2}\right)\frac{\gamma}{\ell_s}. \tag{63}$$

The free energy increase induced by the elastic distortions is given by

$$\Delta F_{el} = \frac{\pi K L_{\rm c}}{4}\sum_{n=1}^{\infty} n b_n^2, \tag{64}$$

which in the linearized regime for small γ gives a simple analytical result

$$\Delta F_{el} = \frac{7KL_{\rm c}}{8\pi^3}\zeta(3)\left(\frac{\gamma}{\ell_s}\right)^2, \tag{65}$$

with $\zeta(3) \approx 1.2$ a constant from the Riemann-Zeta function $\zeta(x)$. The surface anchoring free energy reads

$$F_s = -\frac{L_{\rm c}D_{\rm c}W_0}{2} 2\int_0^{D_{\rm c}} dz \cos^2(\gamma - \varepsilon(0,z)), \tag{66}$$

where the factor two reflects the two opposing sides of the rectangular strip with surface $L_{\rm c}D_{\rm c}$ whose contributions are equivalent. Then, in the absence of elastic distortions and no tilt ($\gamma = 0$) the surface anchoring free energy would simply be $F_s = -L_{\rm c}D_{\rm c}W_0$ which only marginally differs from the result for the cylindrical case $F_s = -(\pi/4)L_{\rm c}D_{\rm c}W_0$. Within the linearized regime for small tilt angles $\gamma \ll 1$ the change in surface anchoring free energy imparted by the elastic distortions is given by

$$\begin{aligned}\Delta F_s &\approx L_{\rm c}D_{\rm c}W_0\int_0^{D_{\rm c}} dz(\gamma - \varepsilon(0,z))^2 \\ &\approx W_0 L_{\rm c}D_{\rm c}\left(1 + \frac{1}{48\ell_s^2} - \frac{7\zeta(3)}{\pi^3\ell_s}\right)\gamma^2.\end{aligned} \tag{67}$$

This expression along with Eq. (65) clearly reflects the basic trade-off between surface anchoring and elasticity where the cost in elastic free energy is partly compensated by a reduction of the surface anchoring free energy (last term between brackets). The total free energy change for small tilt angles now reads

$$\Delta F_{\rm tot} \sim W_0 L_{\rm c}D_{\rm c}\left(1 - \frac{49\zeta(3)}{8\pi^3\ell_s}\right)\gamma^2 + \mathcal{O}(\gamma^2/\ell_s^2). \tag{68}$$

Let us now compare our results with the simple RP expression Eq. (21) in the *absence* of elastic distortions. Taking $\theta = \pi/2$ and expanding for small γ we find

$$\Delta F_{\rm tot}^{(s)} \sim \frac{\pi}{4}W_0 L_{\rm c}D_{\rm c}\gamma^2. \tag{69}$$

Disregarding the trivial curvature prefactor $\pi/4$ in the last expression, we find that the impact of the elastic distortions is rather marginal, since the correction term in Eq. (68) is less than 1 k_BT. Numerical resolution of Eq. (62) reveals that weak elastic distortions occur mostly when the rod is at an oblique angle $\gamma = \pi/4$. The predictions from our analytical model are depicted in Fig. 7(b).

[1] Mundoor, H., Park, S., Senyuk, B., Wensink, H. H. & Smalyukh, I. I. Hybrid molecular-colloidal liquid crystals. *Science* **360**, 768 (2018).

[2] Priest, R. G. & Lubensky, T. C. Biaxial model of cholesteric liquid crystals. *Physical Review A* **9**, 893 (1974).

[3] Vroege, G. J. & Lekkerkerker, H. N. W. Phase transitions in lyotropic colloidal and polymer liquid crystals. *Rep. Prog. Phys.* **55**, 1241 (1992).

[4] Mundoor, H., Wu, J.-S., Wensink, H. H. & Smalyukh, I. I. Thermally reconfigurable monoclinic nematic colloidal fluids. *Nature* **590**, 268 (2021).

[5] Stroobants, A. & Lekkerkerker, H. N. W. Liquid crystal phase transitions in a solution of rodlike and disklike particles. *J. Phys. Chem.* **88**, 3669–3674 (1984).

[6] Herzfeld, J., Berger, A. E. & Wingate, J. W. A highly convergent algorithm for computing the orientation distribution functions of rodlike particles. *Macromolecules* **17**, 1718–1723 (1984).

[7] de Gennes, P. G. & Prost, J. *The Physics of Liquid Crystals* (Clarendon Press, Oxford, 1993).
